# Isolation, Genomic Evolution, and Pathogenicity of *Clostridium perfringens* Type A Causing Hemorrhagic Enteritis in Adult Yaks in Southwest China

**DOI:** 10.3390/ani16152391

**Published:** 2026-08-03

**Authors:** Long Zhao, Yongqiang Miao, Zhen Yang, Kefei Shen, Dengfeng Xu, Suhui Zhang, Liu Yang, Lizhi Fu, Ziqi Li, Bo Lian, Yuandi Yu

**Affiliations:** 1 Chongqing Academy of Animal Sciences, Chongqing 402460, China; zhaolong4480@163.com (L.Z.); miaoyq@cqaa.cn (Y.M.); yangz@cqaa.cn (Z.Y.); coffeeshine@163.com (K.S.); firstfengx@163.com (D.X.); 13667653563@163.com (S.Z.); yangl@cqaa.cn (L.Y.); flzfulizhi@163.com (L.F.); 15723023917@163.com (Z.L.); 2 National Technology Innovation Center for Pigs, Chongqing 402460, China; 3 Rongchang, Animal Disease, Observation and Research Station, Ministry of Agriculture and Rural Affairs, Chongqing 402460, China; 4 College of Veterinary Medicine, Southwest University, Chongqing 400715, China; 18184884496@163.com

**Keywords:** *Clostridium perfringens* type A, yak, hemorrhagic enteritis, whole-genome

## Abstract

*Clostridium perfringens* type A causes neonatal clostridial enteritis, hemorrhagic enteritis, and sudden death syndrome in cattle. This study examined a fatal outbreak at a fattening farm housing 58 adult yaks in Southwest China, with an incidence of 22.41% (13/58) and a 100% case fatality rate. Histopathology of deceased yaks showed widespread multi-organ hemorrhage and severe hemorrhagic enteritis. A specific PCR assay amplified the alpha-toxin gene of *Clostridium perfringens*, while no other pathogens were detected. *Clostridium perfringens* was isolated from small intestinal contents using TSC agar, designated strain CQ1, and identified as type A through toxinotyping. Whole-genome sequencing revealed that strain CQ1 carries the core alpha-toxin gene along with key accessory virulence determinants (*pfoA*, *colA*, and *cloSI*). Phylogenomic analysis based on core single-nucleotide polymorphisms revealed that strain CQ1 is clustered into a distinct, independent evolutionary lineage with an existing historical yak isolate and a canine-origin isolate. Intraperitoneal challenge in mice confirmed its extreme lethality, causing 100% mortality within 36 h with severe intestinal necrosis. These findings provide fundamental genetic resources for understanding the virulence mechanisms and genetic background of this pathogen and offer critical insights for preventing and controlling clostridial diseases in high-altitude yak farming.

## Simple Summary

*Clostridium perfringens* is a critical pathogen associated with sudden death syndrome in cattle. In this study, a *Clostridium perfringens* type A strain, designated CQ1, was isolated from a fatal outbreak in adult yaks. The results of whole-genome sequencing and experimental pathogenicity assays demonstrated that strain CQ1 belongs to an independent evolutionary lineage and possesses a lethal toxin system regulated by a conserved signaling pathway, exhibiting extreme lethality in mice. These findings successfully reveal the genetic background and pathogenic mechanisms of this plateau-adapted strain. This study provides a fundamental theoretical basis for developing specialized vaccines and effective treatment strategies in high-altitude pastoral animal husbandry.

## 1. Introduction

*Clostridium perfringens* (*C. perfringens*) is a critical zoonotic pathogen that poses serious threats to both animal husbandry and public health worldwide [1]. As a ubiquitous anaerobic, spore-forming Gram-positive bacterium, it is widely distributed in soil, sewage, food, and the intestinal tracts of humans and animals, facilitating cross-species contamination of food chains [2]. Its virulence is primarily mediated by the production of multiple toxins, and strains are classified into seven types (A–G) based on the presence of six major toxin genes: *plc/cpa* (alpha-toxin), *cpb* (beta-toxin), *etx* (epsilon-toxin), *iap/ibp* (iota-toxin), *cpe* (enterotoxin), and *netB* (necrotic enteritis toxin B) [3]. Notably, type A strains are the most prevalent globally, defined by the constitutive expression of *plc/cpa* (encoding alpha-toxin, also known as phospholipase C) and the absence of other major toxin-encoding genes [3,4]. Alpha-toxin is a multifunctional phospholipase with hemolytic, necrotizing, and lethal activities and plays a central role in the pathogenesis of enteric and histotoxic infections in various animal species [5].

*C. perfringens* type A is a well-documented etiological agent of severe enteric diseases in cattle, including neonatal clostridial enteritis, hemorrhagic enteritis, and sudden death syndrome (SDS) [3,5,6]. These diseases typically present with acute onset, a short disease course, and high mortality, with SDS often causing calf death within days of symptom onset [7]. The yak (*Bos grunniens*) is a unique bovine species endemic to the Qinghai–Tibet Plateau and adjacent high-altitude regions in Southwest China. In recent years, yak husbandry has been undergoing a structural transition from traditional extensive pastoral grazing to intensive semi-housing systems centered on grazing with dietary supplementation. Although this modern production mode has substantially enhanced the economic benefits of yak farming, the increased proportion of concentrated feed coupled with multi-factorial stressors frequently triggers intestinal microbiota dysbiosis, significantly driving up the incidence of diarrhea and compromising farming efficiency [8,9]. Previous investigations into yak diarrhea have primarily focused on specific viral pathogens such as bovine coronavirus (BCoV), bovine rotavirus (BRV), bovine viral diarrhea virus (BVDV), and bovine torovirus (BToV) [10,11,12,13]. Although the detrimental impacts of *C. perfringens* in yaks are documented [14,15,16], comprehensive investigations into the epidemiological and pathogenic characteristics of this pathogen in yak herds remain scarce. Existing studies indicate that *C. perfringens* isolates circulating in yak populations are predominantly classified as type A [14,16]. Clinical manifestations in affected yaks are characteristically marked by diarrhea, abdominal distension, or intestinal hemorrhage [15], yet detailed pathological features have not been systematically reported. Elucidating its antimicrobial resistance, pathogenicity, and genomic characteristics is urgently needed to guide clinical therapeutics and targeted vaccine development.

In this study, a *C. perfringens* type A strain was successfully isolated from a fatal field outbreak among adult yaks in Southwest China. Subsequent systematic phenotypic and genotypic characterizations of the isolate were performed. Toxinotypes and virulence gene profiles were determined, antimicrobial resistance phenotypes were assessed, and pathogenic potential was evaluated via a murine infection model. These findings provide fundamental genetic resources for understanding the virulence mechanisms and genetic background of this pathogen, offering critical insights for the clinical prevention and control of clostridial diseases in high-altitude yak husbandry.

## 2. Materials and Methods

### 2.1. Farms and Samples

In July 2025, a sudden mortality outbreak occurred in a herd of 58 male yaks (18 to 24 months old) under intensive feedlot management in Southwest China, one week following a forage transition to new supplies. The animals were fed commercial hay and concentrate, with a vaccination history limited to foot-and-mouth disease. Initially, 3 yaks died abruptly without prior clinical signs. Despite reverting to the original feed and implementing farm-wide disinfection, the disease progressed, affecting an additional 10 yaks within one week. Prior to sampling, intramuscular injection of ceftiofur hydrochloride was administered but failed to alleviate symptoms. Overall, the disease reached an incidence rate of 22.41% (13/58), with all affected yaks dying within 1 to 5 days post-onset, resulting in a case fatality rate of 100% (13/13).

Intestinal contents, rectal swabs, nasal swabs and blood samples were collected from dead yaks for pathogen detection and isolation, and visceral tissues were additionally sampled for pathological examination.

### 2.2. Histopathological

Tissue blocks from the lungs, jejunum, liver, spleen, kidneys and heart were collected and fixed in 10% neutral buffered formalin solution (Beijing Solarbio Science & Technology Co., Ltd., Beijing, China). These tissues were processed using a graded alcohol series, cleared in xylene, and embedded in paraffin wax. For each collected organ, three paraffin-embedded tissue sections were prepared and stained with hematoxylin and eosin (H&E). A minimum of five randomly selected microscopic fields per section were examined using an optical microscope to evaluate pathological changes.

### 2.3. Examination of Pathogen

The initial pathogen screening panel (*C. perfringens*, *Pasteurella multocida*, BVDV, BCoV, BRV, and BToV) was selected based on established regional epidemiological evidence in yaks and the acute, sudden death clinical presentation observed in this outbreak [10,11,12,13,14,17]. Specific PCR assays were performed on the small intestinal contents, anal swabs, nasal swabs, and blood samples collected from the dead cattle. DNA and RNA were extracted from these samples using a commercial kit following the manufacturer’s instructions (Tiangen Biotech Co., Ltd., Beijing, China). The RNA was further reverse-transcribed into complementary DNA (cDNA) (Takara Bio, Kusatsu, Japan). The specific PCR was performed to detect six toxins of *C. perfringens* as described in previous studies [3]. To ensure the specificity and accuracy of the toxin typing in the absence of standard reference strains, all target PCR-amplified bands were excised from agarose gels, purified using a DNA Gel Extraction Kit (TIANGEN Biotech Co., Ltd., Beijing, China), and sequenced by Sangon Biotech Co., Ltd. (Chengdu, China). The resulting sequences were verified against the GenBank database using BLASTn (https://blast.ncbi.nlm.nih.gov/Blast.cgi accessed on 26 July 2026) to confirm target toxin gene identities. Meanwhile, PCR amplifications were specifically conducted for *Pasteurella multocida* [18], BVDV [13], BCoV [19], BRV [20], and BToV [12], respectively. Detailed primer information for these pathogens is provided in Supplementary Table S1.

### 2.4. Bacterial Isolation and Identification

Samples were inoculated into Anaerobic Meat Liver Broth (Beijing Solarbio Science & Technology Co., Ltd., Beijing, China) and incubated anaerobically at 37 °C for 12 h. Cultures were then streaked onto Tryptose Sulfite Cycloserine (TSC) agar and 5% sheep blood agar (HopeBio, Qingdao, China). Isolated colonies were subjected to Gram staining and *16S rRNA* gene sequencing for species confirmation. The *16S rRNA* gene was amplified using universal primers [21]. Purified amplicons were Sanger-sequenced, edited in SnapGene, and compared against the NCBI database using BLASTn. Phylogenetic analysis was performed using the neighbor-joining method in MEGA 11 with 1000 bootstrap replicates.

### 2.5. Antimicrobial Susceptibility Testing

Antimicrobial susceptibility was determined by the disc diffusion method using commercial antibiotic discs (Hangzhou Microbi, Hangzhou, China). Bacterial suspensions adjusted to a 0.5 McFarland standard were uniformly swabbed onto Mueller-Hinton agar supplemented with 5% defibrinated sheep blood, and the discs were applied prior to anaerobic incubation at 37 °C for 18 h. *C. perfringens* ATCC 13124 was utilized as the quality control strain for all testing procedures. Inhibition zone diameters were measured in triplicate using independent biological replicates, and susceptibility results were interpreted as susceptible, intermediate, or resistant according to the Clinical and Laboratory Standards Institute guidelines (CLSI M100-Ed32).

### 2.6. Whole-Genome Sequencing and Bioinformatic Analysis

Whole-genome sequencing of *C. perfringens* was performed using the Illumina PE150 and PacBio Sequel systems. Genome assembly was performed using Hifiasm and Canu, with Pilon used for sequence polishing. Assembly completeness and integrity were evaluated using BUSCO. Plasmid identification was conducted using PlasmidFinder and BLASTn searches against the NCBI plasmid database. Protein-coding genes and non-coding RNAs were predicted using Prodigal and tRNAscan-SE, respectively. Functional annotation was conducted against NCBI, GO, KEGG, COG, and VFDB databases. Antimicrobial resistance genes and virulence factors were screened via BLAST (version 2.3.0) against the CARD and VFDB databases, with an E-value cutoff ≤ 10^−5^.

To evaluate genetic relationships, 45 representative *C. perfringens* complete genomes from diverse host species were retrieved from the NCBI database (Supplementary Table S2). Core Single Nucleotide Polymorphisms (SNPs) across all strains were identified using Snippy (v4.6.0) under default parameters with *C. perfringens* (GCF_000171195.1) serving as the reference genome. Recombination filtering was not applied during SNP processing; consequently, phylogenetic distances are interpreted as metrics of overall genetic relatedness rather than direct transmission dynamics. Based on the calculated pairwise SNP distance matrix, a minimum spanning tree (MST) was constructed using GrapeTree (v1.5.0) and visualized via iTOL (https://itol.embl.de/).

### 2.7. Animal Virulence Assay

To evaluate pathogenicity, ten female specific-pathogen-free Kunming mice (18–22 g, 6–8 weeks old; Hunan Slack Jingda Laboratory Animal Co., Changsha, China) were randomly allocated into two groups (*n* = 5) following a one-week acclimatization period. The isolated *C. perfringens* strain was cultured in Anaerobic Meat Liver Broth to late log phase, harvested by centrifugation (4000× *g*, 10 min), and resuspended in sterile PBS to a concentration of 1 × 10^8^ CFU/mL. Mice in the test group were injected intraperitoneally with 0.5 mL of the bacterial suspension, while the control group received an equal volume of sterile PBS. Animals were monitored twice daily for 2 days for predefined humane endpoints (e.g., severe lethargy, dyspnea). Moribund and surviving mice were euthanized via cervical dislocation under deep anesthesia induced by sodium pentobarbital (150 mg/kg) (Sigma-Aldrich, St. Louis, MO, USA), followed by immediate necropsy and tissue collection for histopathology.

### 2.8. Statistical Analysis

Statistical analyses were performed using GraphPad Prism V8.0. For the animal virulence assay, survival curves between the control and infected groups were compared using the log-rank (Mantel–Cox) test. A two-tailed *p* < 0.05 was considered statistically significant.

## 3. Results

### 3.1. Clinical Signs

The affected yaks exhibited a rapid disease progression, with the shortest time from symptom onset to death being approximately 10 h. The main clinical manifestations included watery diarrhea with significant dark red blood (Figure 1A), abdominal distension, blood-tinged nasal discharge oozing from the nostrils (Figure 1B) and dyspnea. Some affected cattle also showed swelling of the forelimb joints, and anal protrusion and discharge of bright red blood from the anus (Figure 1C). Shortly after death, post-mortem examination was conducted. The mucosa of the small intestines was red due to severe hemorrhage and the intestinal lumen was filled with reddish contents (Figure 1D). Numerous petechial hemorrhages were observed in the lungs, heart, kidneys, and spleen (Figure 1E–H). No other abnormalities were found in the carcass.

### 3.2. Histopathological Lesions of Deceased Yaks

Histopathological examination revealed the following lesions in various organs. In lung tissue, alveolar lumina were filled with abundant serous fluid, the normal alveolar architecture was disrupted, and extensive hyperemia was also observed in the lung parenchyma (Figure 2A). Jejunal tissue exhibited intestinal villus necrosis, accompanied by extensive sloughing of epithelial cells from the lamina propria and widespread hemorrhage within the submucosal tissue (Figure 2B). Hepatocyte exhibited swelling, along with hemorrhage at the margins of hepatic lobules in liver tissue (Figure 2C). Spleen tissue presented massive infiltration of red blood cell in the red pulp region, along with dense petechiae (Figure 2D). Widespread hemorrhage was observed in the renal cortex and medulla (Figure 2E). Cardiac tissue displayed severe myocardial hemorrhage, accompanied by necrosis of cardiomyocytes and myocardial fiber rupture (Figure 2F). Collectively, these histopathological features demonstrate that the pathological basis in this acutely deceased yak is anchored by severe multi-organ vascular damage, widespread parenchymal hemorrhage, and acute tissue necrosis (particularly in intestinal villi and myocardium), providing direct pathological evidence of fatal systemic toxemia.

### 3.3. Examination of Pathogen

The PCR results were positive for the alpha-toxin gene of *C. perfringens* in all samples, and negative for all other pathogens (Figure 3A). Sanger sequencing and BLAST analysis confirmed that the amplified fragment was 100% identical to the alpha-toxin gene, whereas the minor bands observed for other toxin primers were confirmed to be non-specific amplification sequences, thereby validating the PCR-based toxin typing of the isolate as type A. Gram staining revealed a large number of Gram-positive bacilli in both blood and small intestinal contents, arranged singly or in pairs (Figure 3B).

### 3.4. Bacterial Isolation and Identification

Following anaerobic incubation at 37 °C for another 24 h, black, circular, and flat colonies were observed on the TSC agar (Figure 3C). Gram staining of these colonies revealed numerous of Gram-positive bacilli stained blue-purple (Figure 3D).

The *16S rRNA* gene sequences (GenBank accession No. PX724095) were aligned against the reference sequences of *C. perfringens* strains in the NCBI database, revealing 100% identity. Phylogenetic analysis showed that the isolated strain was most closely related to avian-derived *C. perfringens* strains (Figure 3E). Additionally, only the alpha-toxin gene was detected in all isolated strains; other toxin genes were not detected. Based on the criteria of *C. perfringens* toxinotyping, the clinical manifestations of the affected cattle, and histopathological lesions, the final diagnosis was confirmed as *C. perfringens* type A infection.

### 3.5. Antimicrobial Susceptibility Testing

Antimicrobial susceptibility testing revealed that *C. perfringens* strain CQ1 possessed resistance to multiple antimicrobial agents (Table 1). The isolate demonstrated resistance (R) to sulfamethoxazole-trimethoprim, tetracycline, and doxycycline, and exhibited intermediate susceptibility (I) to cefalexin and minocycline. In contrast, the strain was susceptible (S) to enrofloxacin, ampicillin, carbenicillin, penicillin, piperacillin, cefoperazone, and ceftriaxone.

### 3.6. Genome Sequencing and Analysis

The genome sequence of the *C. perfringens* isolate, designated as CQ1, was deposited in GenBank under the BioProject accession number PRJNA1473206. This assembly yielded an estimated genome size of 3,091,806 bp with a GC content of 28.47% (Supplementary Figure S1). The hybrid assembly produced a complete circular chromosome of 3,091,806 bp with an N50 value of 3.09 Mb, an average sequencing depth of 100.60×, and a genome coverage of 88.48%. No plasmid sequence was identified in this strain. Complete genomic annotation datasets, including all annotated virulence genes, resistance genes, and metabolic pathways, are provided in Supplementary File S1. The minimum spanning phylogenetic tree of the evaluated *C. perfringens* strains is illustrated in Figure 4A. The newly isolated strain, CQ1, demonstrated a close evolutionary relationship with the Chinese yak-origin isolate GCF_022429605.1 and the Russian canine-origin isolate GCA_003990265.1. Notably, these three strains all belong to type A and clustered together to form a distinct, independent evolutionary lineage that is phylogenetically divergent from the other reference strains, indicating that it represents a genetically distinct lineage.

To further quantify this genomic divergence, a pairwise SNP analysis was performed against the 45 reference *C. perfringens* genomes. The SNP distances between strain CQ1 and the 45 reference strains varied significantly, with the maximum distance exceeding 50,000 single-nucleotide polymorphisms (SNPs). Consistent with the topology of the phylogenetic tree, strain CQ1 exhibited substantially lower SNP variations when compared to the Chinese yak isolate GCF_022429605.1 and the Russian canine isolate GCA_003990265.1 than to the remaining 43 reference strains (Figure 4B).

### 3.7. Virulence and Antibiotic Resistance Gene Profiling

WGS analysis identified the *C. perfringens* isolate as type A, harboring the core alpha-toxin gene *plc* (97.5% identity) while lacking *cpb*, *etx*, *iap*, *cpe*, and *netB*. Additionally, several accessory virulence genes were detected with high homology, including *pfoA* (perfringolysin O), *colA* (collagenase), and the protease gene *cloSI*. The presence of a complete type IV pilus system (e.g., *pilB/C/D*) and sialidase genes (*nanH/I*) further underscores the isolate’s potential for tissue colonization and invasive pathogenesis (Table 2).

Key resistance determinants included high-homology efflux pump genes *tetA(P)* (97.8% identity) and *tet(38)* conferring tetracycline resistance. The *mprF* gene (97.2% identity), associated with resistance to cationic antimicrobial peptides, was also identified. Furthermore, the resistome encompassed genes linked to resistance against aminoglycosides (*rpsL*), streptogramins (*vatF*), and sulfonamides (*sul4*). Other detected determinants included the *efrA/B* efflux system and *gyrA/B* mutations, correlating with the observed or potential resistance to macrolides and fluoroquinolones (Table 3).

### 3.8. Pathogenicity in a Mouse Model

All mice in the negative control group survived and remained healthy throughout the 36-h experimental period. In contrast, mice challenged with the isolate reached 100% mortality within 36 h post-injection (Figure 5A). Gross necropsy of the deceased mice revealed intestinal liquefaction, with the lumens filled with yellow contents (Figure 5B). Histopathological analysis demonstrated severe lesions in the small intestine, characterized by congestion, mucosal degeneration, and necrosis with extensive sloughed epithelial cells (Figure 5C).

### 3.9. Outcome and Follow-Up

Based on the rapid diagnostic outcomes and antimicrobial susceptibility testing, penicillin-class antibiotics were promptly administered to the clinically affected cattle for targeted therapy. Concurrently, healthy animals in the herd were immunized with a Clostridium perfringens-specific toxoid vaccine as a preventive measure. Within one week following the implementation of these interventions, no new cases or mortalities were recorded, indicating effective control of the outbreak. The swift clinical recovery following targeted antimicrobial therapy and the blockade of transmission via type-specific vaccination further provide strong therapeutic and epidemiological verification of *C. perfringens* strain CQ1 as the primary etiological agent.

## 4. Discussion

*C. perfringens* is a ubiquitous pathogen that induces a diverse spectrum of enterotoxemic diseases, mastitis, severe diarrhea, and sudden death across livestock species [6,22,23]. Furthermore, its widespread contamination of milk and meat products poses a persistent threat to public health and food safety [4,24]. Worldwide, type A strains are recognized as the primary genotypic drivers of bovine diarrhea, necro-hemorrhagic enteritis, and sudden death [6,22,25,26,27]. Recent epidemiological investigations in China focusing on ruminants as well as contaminated milk and meat products similarly confirm that type A remains the predominant genotype [24,28]. Crucially, studies in domestic yak populations also establish type A as the dominant circulating genotype [14,16], which directly aligns with the isolates identified in the present study. Establishing this clear epidemiological baseline provides an essential scientific rationale for formulating targeted vaccine strains and immunization strategies tailored to yak health on the plateau.

Here we characterize a *C. perfringens* isolate recovered from adult yaks, integrating microbiological, genomic, and experimental virulence analyses to reveal new insights into the pathogen’s genetic composition and pathogenicity in this distinctive ruminant. Published reports regarding diarrhea, enteritis, hemorrhagic enteritis, and sudden death in yaks remain notably scarce, with existing literature predominantly focused on the isolation and genomic variation of pathogens such as BRV, BCoV, and BVDV [10,11,13]. While previous studies have isolated *C. perfringens* type A from yaks suffering from severe diarrhea, abdominal distension, and intestinal hemorrhage, the specific pathological features associated with these field cases remained uncharacterized [15]. To our knowledge, this study provides the first systematic integration of clinical manifestations and detailed necro-hemorrhagic pathology of *C. perfringens* type A infection in yaks, establishing an essential pathological diagnostic foundation and critical epidemiological insights for managing clostridial diseases in yaks.

The primary etiology of this fatal outbreak was confirmed as *C. perfringens* type A via exclusive PCR detection of the alpha-toxin gene and successful bacterial isolation, ruling out common viral co-infections. The fulminant progression and severe necro-hemorrhagic enteritis observed in these adult yaks differ from typical domestic cattle infections [6]. While host eco-physiological characteristics unique to high-altitude ruminants might contribute to baseline differences in mucosal immunity or stress responsiveness [29], the precise role of high-altitude adaptation requires dedicated comparative genomic and physiological validation. Instead, the abrupt dietary shift to intensive semi-housing rations likely served as a major critical stressor that induced intestinal microbiota dysbiosis. This physiological perturbation disrupts mucosal barrier integrity [30], rendering the yak gastrointestinal tract highly vulnerable to *C. perfringens* proliferation. Consequently, the produced alpha-toxin, acting as a multifunctional phospholipase C, hydrolyzes phosphatidylcholine and sphingomyelin on host cell membranes, triggering cytolysis, extensive hemolysis, and severe vascular endothelial damage [5]. This enzymatic disruption degrades vascular wall integrity, facilitating systemic toxin translocation and driving the rapid progression toward multi-organ failure and systemic hemorrhage.

Phylogenetic analysis based on SNPs showed that *C. perfringens* strain CQ1 belongs to a distinct, independent evolutionary lineage. Strain CQ1 showed the closest genetic proximity to a historical yak isolate determined in the Chinese Qinghai region (GCF_022429605.1), indicating that *C. perfringens* strains in yak within this lineage showed unique evolutionary characteristics. The unique evolution of *C. perfringens* in yak may be related to the host species and special geographical environment of the Qinghai–Tibet Plateau, i.e., the high-altitude, low oxygen, and extreme diurnal temperature fluctuations [31]. In fact, this unique evolutionary trend associated with the yak host has been widely observed in various other pathogens previously, as evidenced by the distinct lineages formed by BRV, BCoV, and BToV [10,11,12]. Interestingly, a Russian canine-origin isolate (GCA_003990265.1) was also located at this identical lineage. While this phylogenetic topology suggests a potential evolutionary link or shared lineage between strains from ruminants and companion animals, further epidemiological studies are required to determine whether cross-species transmission actually occurs. Therefore, comprehensive genomic surveillance across intersecting ecosystems is warranted to monitor the evolutionary dynamics and potential risks of this genetically distinct lineage.

The antimicrobial susceptibility profiles of *C. perfringens* strain CQ1 demonstrated a highly aligned yet nuanced correlation with its genomic resistome background. A high degree of phenotypic-genotypic concordance was observed for tetracyclines and sulfonamides, as absolute resistance to tetracycline, doxycycline, and sulfamethoxazole-trimethoprim was robustly underpinned by the presence of corresponding resistance determinants such as *tetA(P)* and *sul4*. Conversely, a notable discordance occurred regarding fluoroquinolones, where the strain remained phenotypically susceptible to enrofloxacin despite harboring a *gyrA* mutation, likely due to low transcriptional efficiency or a mutation outside the canonical quinolone resistance-determining region [32,33]. Crucially, the strain remained highly susceptible to penicillin antibiotics, which is consistent with a previous report, indicating that penicillin remains the drug of choice for the clinical treatment of *C. perfringens* infections in yaks.

Beyond its resistome, strain CQ1 harbors the core alpha-toxin gene alongside accessory toxin genes (*pfoA*, *colA*, and *cloSI*). In *C. perfringens*, the expression of such lethal factors is orchestrally governed by the *VirS/VirR* system. Specifically, upon sensing Agr-mediated signaling peptides, *VirS/VirR* triggers a signaling cascade involving regulatory RNAs (such as VR-RNA and virU) to directly or indirectly activate the transcription of toxin genes including *pfoA* [34,35]. This efficient and coordinated expression of virulence genes likely constitutes an important molecular foundation for the virulent phenotype observed in strain CQ1. To rapidly evaluate this acute toxicity, intraperitoneal inoculation was employed in the mouse model to ensure consistent bioavailability of the bacterial suspension and secreted toxins [14], avoiding variable oral colonization caused by host microbiota resistance. Although this model bypasses gastric acid barriers, mucosal immunity, and microbial competition inherent to natural oral infection, the distinct phenotypic contrast (0% vs. 100% 36-h mortality) with n = 5 per group provided statistical power exceeding 90% in the Log-rank test (*p* < 0.001), ensuring statistical validity while satisfying the 3Rs reduction principle. This lethal toxic dynamic directly explains the acute fatality of the adult yak field case, which correlates perfectly with the experimental pathogenesis in mice. Consistent biological characteristics observed between natural lethal infection and experimental pathogenesis demonstrate that strain CQ1 serves as an ideal model to investigate virulence regulatory mechanisms in hypervirulent *C. perfringens*.

*C. perfringens* type A is widely present as a gut commensal, making its definitive pathogenic role in enteric diseases challenging to establish. Epidemiologically, the onset of the outbreak was secondary to a sudden feed change, which represents a classic predisposing trigger capable of altering the intestinal microenvironment to promote rapid proliferation and toxin synthesis of *C. perfringens*. Clinically, affected yaks exhibited peracute signs, including severe bloody diarrhea and abdominal bloat, leading to rapid death within 10 h. Correspondingly, gross necropsy and histopathological examinations revealed severe necro-hemorrhagic enteritis accompanied by widespread multi-organ hemorrhages. These pathological manifestations reflect extensive endothelial damage, which is consistent with the synergistic histotoxicity and cytotoxicity of alpha-toxin and *pfoA* reported in previous studies on *C. perfringens* [5]. Genomic analysis of strain CQ1 unveiled a potent virulence architecture, including the core alpha-toxin gene and accessory toxin genes (*pfoA*, *colA*, *cloSI*). Crucially, a post-outbreak follow-up survey demonstrated that targeted administration of penicillin-class antibiotics combined with toxoid vaccination eradicated new cases within one week, providing confirmatory therapeutic evidence. Collectively, these lines of evidence strongly identified strain CQ1 as the primary pathogen responsible for this acute outbreak in adult yaks (Figure 6).

Nevertheless, several limitations of this study should be acknowledged. Although *Clostridium perfringens* strain CQ1 was isolated in high abundance as the sole predominant pathogen from visceral tissues and intestinal contents, comprehensive screening for other acute enteric pathogens, such as *Mycobacterium avium* subsp. *paratuberculosis*, *Salmonella* spp., *Eimeria* spp., and *Cryptosporidium*, was not routinely performed via targeted selective culture or molecular assays. Although these pathogens typically affect younger calves and rarely induce peracute bloat and sudden death syndrome in adult yaks, their potential contribution as minor co-infections cannot be completely ruled out. Furthermore, whether the mere genomic presence of these virulence genes directly confers high pathogenicity to the strain requires further experimental investigation. Future outbreak investigations should employ broader multi-pathogen diagnostic panels, including metagenomic sequencing, to fully elucidate potential polymicrobial dynamics in high-altitude enteric diseases.

## 5. Conclusions

We successfully isolated a *C. perfringens* type A strain from adult yaks, which clusters into a distinct, independent evolutionary lineage alongside historical yak isolates. These findings expand the current knowledge regarding the genomic evolution of *C. perfringens* in ruminants, thereby providing critical insights for the clinical prevention and targeted control of clostridial diseases in high-altitude yak husbandry.

## Figures and Tables

**Figure 1:**
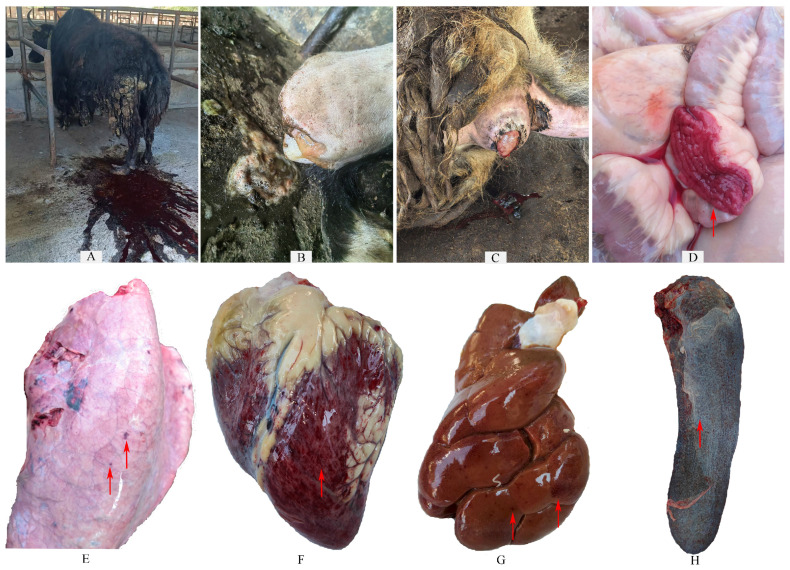
Clinical signs and pathological changes of visceral organs in affected yaks, characterized by widespread multi-organ hemorrhage and severe systemic lesions. (**A**) Hemorrhagic diarrhea with watery consistency. (**B**) Exudation of a mixture of blood and mucus from the nostrils. (**C**) Anal protrusion with hemorrhagic discharge. (**D**) Hemorrhagic mucosa of the small intestine. (**E**) Pulmonary congestion. (**F**) Massive cardiac hemorrhage. (**G**) Renal hemorrhage. (**H**) Splenic hemorrhage. Red arrows indicate hemorrhagic foci.

**Figure 2:**
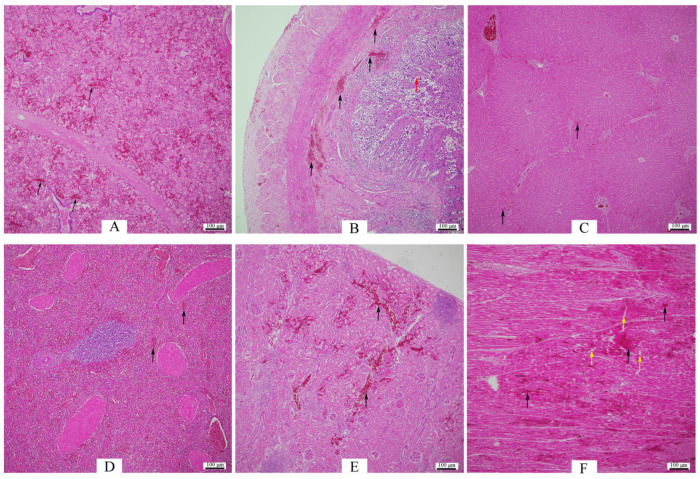
Histopathological changes of internal organs of affected yaks examined using hematoxylin and eosin staining (H&E). (**A**) Lung: Alveolar lumina distended by serous fluid with pulmonary parenchymal hyperemia. (**B**) Jejunum: Extensive submucosa hemorrhage with villus epithelial cells detachment. (**C**) Liver: Perilobular congestion in the hepatic lobules. (**D**) Spleen: Infiltration of red blood cell in the red pulp. (**E**) Kidney: Extensive hemorrhage. (**F**) Heart: Severe myocardial hemorrhage with fiber rupture. The black arrows indicate hemorrhagic sites; the red arrows indicate epithelial cell detachment; and the yellow arrows indicate myocardial fiber rupture.

**Figure 3:**
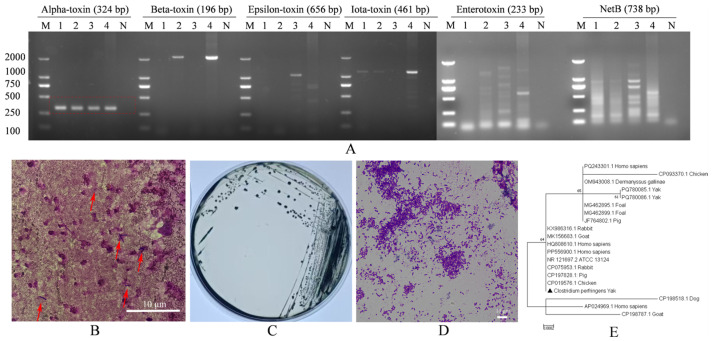
Identification of *C. perfringens* isolated from yak samples. (**A**) Agarose gel electrophoresis results of PCR products for six toxin genes of *C. perfringens*, showing specific amplification exclusively for the alpha-toxin gene (*plc*, 324 bp) with no specific bands for other toxin genes. Lane (M): DNA marker DL 2000; Lane (1–4): small intestinal contents, rectal swabs, nasal swabs, blood (respectively); Lane (N): negative control (ddH_2_O). (**B**) Gram-stained preparation of small intestinal contents showing characteristic Gram-positive bacilli (1000×). Red arrows indicate these bacilli. (**C**) Selective colony morphology of bacterial isolates on TSC agar displaying typical black, circular, and flat colonies. (**D**) Gram–stained preparation of single colonies confirming pure cultures of Gram-positive bacilli (1000×). (**E**) Phylogenetic analysis of *16S rRNA* gene sequences, demonstrating 100% identity and close evolutionary relationship to reference *C. perfringens* strains.

**Figure 4:**
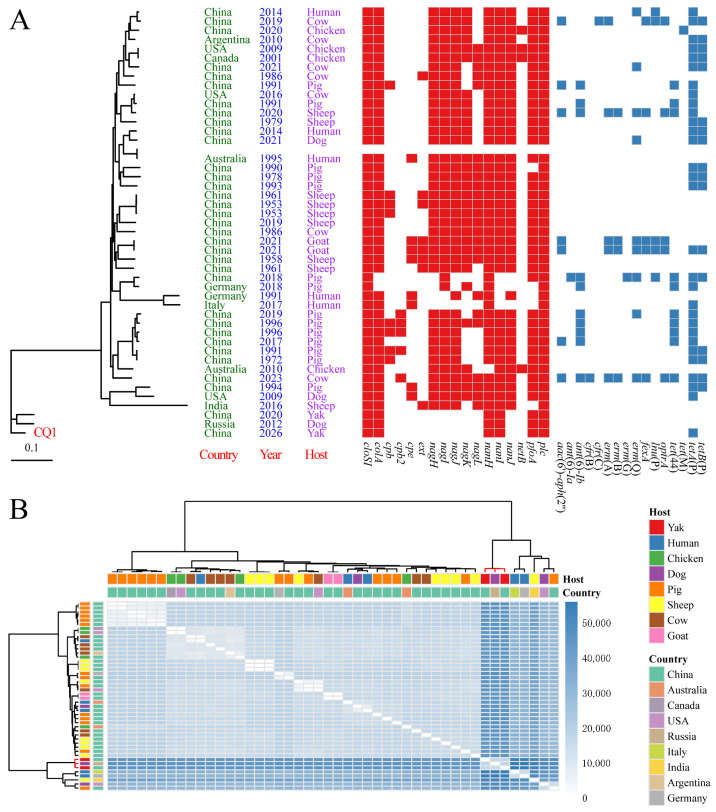
Bioinformatic analysis of whole-genome sequences of the isolates. (**A**) Minimum spanning tree of the 46 *C. perfringens* isolates. Isolates in this study were marked with red color. (**B**) Clustering heatmap based on SNPs in different *C. perfringens* isolates.

**Figure 5:**
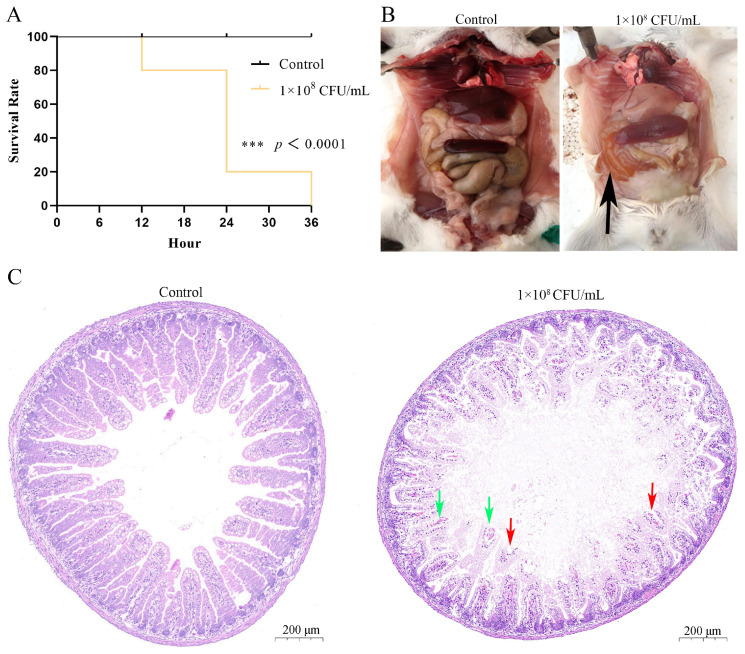
Murine pathogenicity model of *C. perfringens* strain CQ1. (**A**) Cumulative survival curves of the CQ1-challenged group versus the negative control group (*p* < 0.0001, log-rank Mantel–Cox test). (**B**) Gross lesions displaying normal major visceral organs, with pathological changes selectively restricted to small intestinal liquefaction and wall thinning (black arrows). (**C**) Histological view of the small intestine characterized by villus blunting, fusion, and fragmentation (red arrows) with widespread congestion (green arrows).

**Figure 6:**
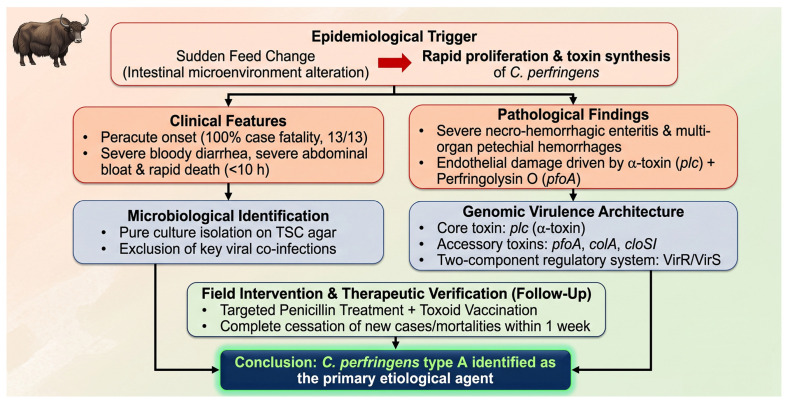
Integrated weight-of-evidence framework establishing the causal role of *C. perfringens* type A in the peracute yak enterotoxemia outbreak.

**Table 1:** Antimicrobial susceptibility phenotypes of *C. perfringens*.

Antibiotic Class	Antibiotic Agent	Result
Folate Pathway Inhibitors	Trimethoprim-Sulfamethoxazole	R
Fluoroquinolones	Enrofloxacin	S
Tetracyclines	Doxycycline	R
	Tetracycline	R
	Minocycline	I
Penicillins	Ampicillin	S
	Carbenicillin	S
	Penicillin	S
	Piperacillin	S
Cephalosporins	Cefoperazone	S
	Cefalexin	I
	Ceftriaxone	S

R—resistant, S—susceptible, I—intermediate.

**Table 2:** Primary virulence genes identified in *C. perfringens* strain CQ1.

Virulence Category	Virulence Factor	Associated Gene
Exotoxins	Phospholipase C (Alpha-toxin)	*plc*
	Perfringolysin O	*pfoA*
	Hemolysins	*hlyB*, *CPF_1725*, *CPF_2072*, *hlyIII*
	Enterotoxins	*entD*, *entB*
Exoenzymes	Collagenase (Kappa-toxin)	*colA*
	Clostripain (Alpha-clostripain)	*cloSI*
	Sialidases (Exo-alpha-sialidase)	*nanL*, *nanH*
Adhesion and Motility	Type IV pili system components	*pilT*, *pilC*, *pilB*, *CPE2281*, *pilN*, *pilM*, *pilC2*, *pilB2*, *pilD*, *pilA1*
	Fibronectin-binding proteins	*fbpA*, *fbpB*
	Streptococcal plasmin receptor/GAPDH	*plr/gapA*
Regulation and Others	VirR/VirS two-component system	*virR*, *virS*
	Chaperonin (60 kDa)	*groEL*
	Elongation factor Tu	*tuf*

**Table 3:** Primary antimicrobial resistance genes identified in *C. perfringens* strain CQ1.

Resistance Category	Product	Associated Gene	Identity (%)
Tetracyclines	Tetracycline resistance active efflux pump (Class P)	*tetA(P)*	97.8
	Tetracycline resistance efflux protein	*tet(38)*	51.4
	Ribosomal protection protein	*rpsJ*	62.7
Folate Pathway Inhibitors	Altered dihydropteroate synthase (Sulfonamide resistance)	*sul4*	50.0
Fluoroquinolones & Macrolides	ABC multidrug efflux pump system components	*efrA/efrB/macB*	57.4/56.2/47.1
	DNA gyrase subunit A mutation	*gyrA*	63.7
Peptides & Lipo-peptides	Lysyl-phosphatidylglycerol synthase (Defensin resistance)	*mprF*	97.2
Aminoglycosides	Ribosomal protein S12 (Streptomycin resistance mutation)	*rpsL*	74.0
Streptogramins	Streptogramin A acetyltransferase	*vatF*	55.6
Fusidic Acid	Elongation factor G mutation/Efflux system	*fusA/fusE*	64.3/62.0

## Data Availability

The sequences reported in this study were submitted to GenBank under accession numbers PX724095 and BioProject accession number PRJNA1473206.

## Supplementary Materials

The following supporting information can be downloaded at: https://www.mdpi.com/article/10.3390/ani16152391/s1, Table S1: Primers used for PCR amplification; Table S2: Forty-five representative complete genomes of *C. perfringens* strains; Figure S1: Genomic information of *C. perfringens* strain CQ1 isolated from yak; File S1: Gene functional annotation, virulence factor and antimicrobial resistance gene prediction of strain CQ1.

## Abbreviations

The following abbreviations are used in this manuscript:

BCoV	Bovine Coronavirus
BToV	Bovine Torovirus
BVDV	Bovine Viral Diarrhea Virus
BRV	Bovine Rotavirus
cDNA	Complementary DNA
*C. perfringens*	*Clostridium perfringens*
H&E	Hematoxylin and eosin
SDS	Sudden Death Syndrome
SNPs	Single Nucleotide Polymorphisms
TSC	Tryptose Sulfite Cycloserine
