# Supplementary material for: Isolation, Genomic Evolution, and Pathogenicity of Clostridium perfringens Type A Causing Hemorrhagic Enteritis in Adult Yaks in Southwest China
Source: Animals (Basel). 2026 Aug 3;16(15):2391. doi: 10.3390/ani16152391 (PMC13464847; doi:10.3390/ani16152391)
Supplement: Supplementary file 1 [file animals-16-02391-s001.zip › Figure S1.pdf]

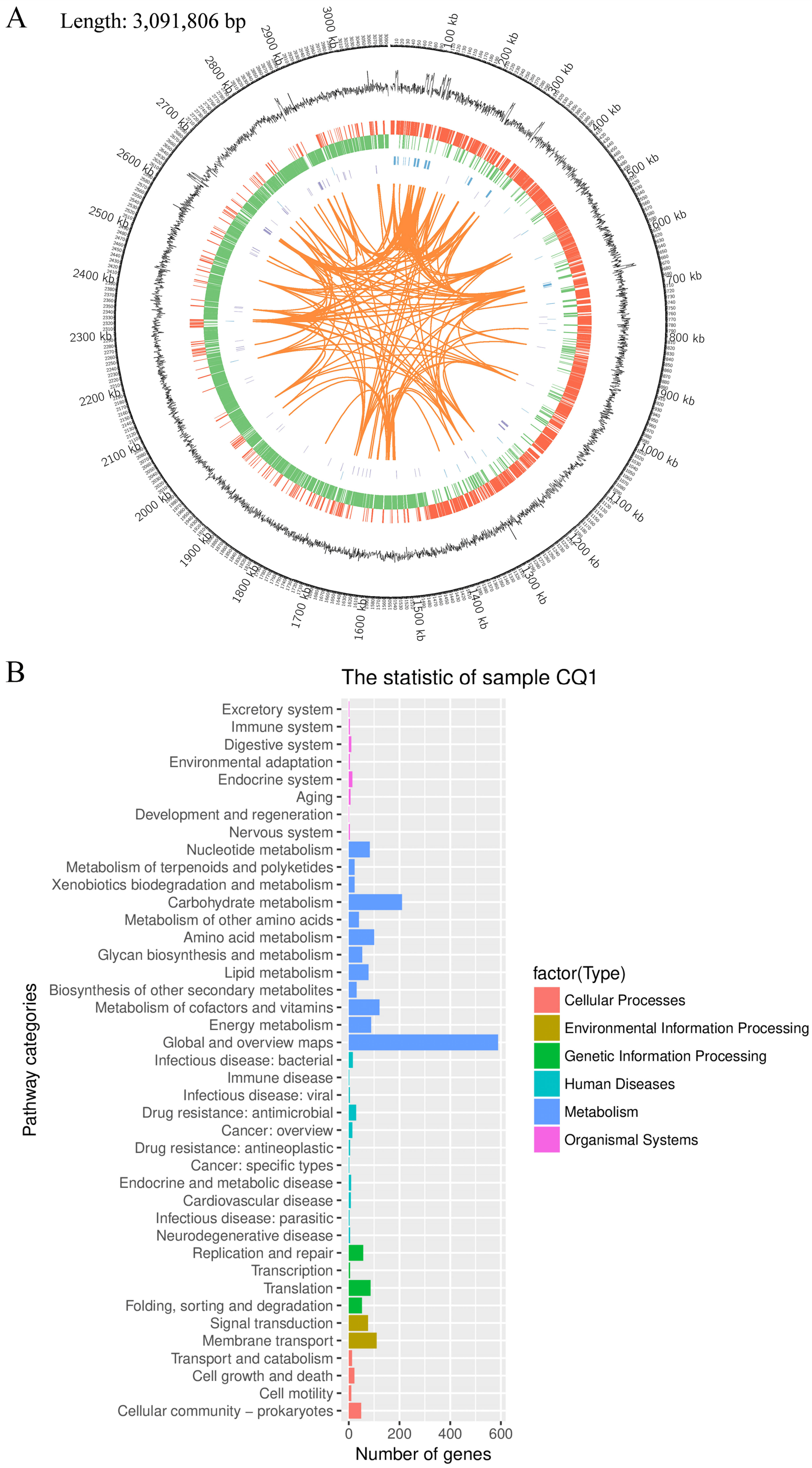

Figure S1. Genomic information of *C. perfringens* strain CQ1 isolated from yak. (A) Circular genome map of *C. perfringens*. From the outermost to the innermost, seven circles are displayed as follows: circle 1 indicates

genomic position information; circle 2 shows GC content; circle 3 represents coding genes on the positive strand (red); circle 4 represents coding genes on the negative strand (green); circle 5 shows ncRNA on the positive strand (blue); circle 6 shows ncRNA on the negative strand (purple); and circle 7 indicates long repeat sequences in the genome (orange). (B) KEGG enrichment analysis of *C. perfringens* strain CQ1.
