# Supplementary material for: Isolation, Genomic Evolution, and Pathogenicity of Clostridium perfringens Type A Causing Hemorrhagic Enteritis in Adult Yaks in Southwest China
Source: Animals (Basel). 2026 Aug 3;16(15):2391. doi: 10.3390/ani16152391 (PMC13464847; doi:10.3390/ani16152391)
Supplement: Supplementary file 1 [file animals-16-02391-s001.zip › Table S1.pdf]

Table S1. Primers used for PCR amplification

Target gene	Primer name	Sequence (5′-3′)	length (bp)	Reference
netB	JRP6656	CTTCTAGTGATACCGCTTCAC	738	
	JRP6655	CGTTATATTCACTTGTTGACGAAAG		
etx	JRP4234	CCACTTACTTGTCCTACTAAC	653	
	JRP4235	GCGGTGATATCCATCTATTC		
iap	JRP5507	GGAAAAGAAAATTATAGTGATTGG		
	JRP5508	CCTGCATAACCTGGAATGGC	461	[3]
plc	JRP4232	GCTAATGTTACTGCCGTTGACC	324	
	JRP4233	CCTCTGATACATCGTGTAAG		
cpe	JRP5179	GGAGATGGTTGGATATTAGG	233	
	JRP5180	GGACCAGCAGTTGTAGATA		
cpb	JRP5181	GCGAATATGCTGAATCATCTA	196	
	JRP5182	GCAGGAACATTAGTATATCTTC		
Pasteurella multocida kmt1	KMT1T7	ATCCGCTATTTACCCAGTGG	460	[16]
	KMT1SP6	GCTGTAAACGAACTCGCCAC		
BCoV-Nsp10	BCoV-F	CGAGTTGAACACCCAGAT	230	[17]
	BCoV-R	CCAARTTYTAYGGHGGITGG		
BToV-M	BToV-F	GCTTGTTGCACTTGATRTTA	114	[12]
	BToV-R	GCTGTTAATGGTTTATTGC		
BRV-VP6	BRV-F	AGAATACATAGAGAACTGGAAT	96	[18]
	BRV-R	GTGAATGACGCTGAATAAG		
BVDV-5′UTR	BVDV-F	GCCATGCCCTTAGTAGGACT	230	[13]
	BVDV-R	CACCCTATCAGGCTGTRTYC		
16S rRNA	16S-F	AGAGTTTGATCCTGGCTCAG	1500	[19]
	16S-R	GGTTACCTTGTTACGACTT		
